# Training experience across UK medicine: deanery differences versus site variation—General Medical Council National Training Survey 2025 analysis

**DOI:** 10.3389/fmed.2026.1833862

**Published:** 2026-06-24

**Authors:** Arkadeep Dhali, Saikat Mandal, Guruprasad Aithal, David S. Sanders

**Affiliations:** 1 Academic Unit of Gastroenterology, Sheffield Teaching Hospitals NHS Foundation Trust, Sheffield, United Kingdom; 2 School of Medicine and Population Health, University of Sheffield, Sheffield, United Kingdom; 3 School of Medicine, University of Nottingham, Nottingham, United Kingdom; 4 NIHR Nottingham Biomedical Research Centre, Nottingham University Hospitals NHS Trust and the University of Nottingham, Nottingham, United Kingdom

**Keywords:** clinical learning environment, deanery, National Training Survey, postgraduate medical education, quality, training, variation

## Abstract

**Background:**

The General Medical Council’s (GMC) National Training Survey (NTS) is widely used to benchmark postgraduate training experience in the UK. However, for actionable interpretation, it is necessary to distinguish deanery-level patterns from variation that is more locally specific to individual sites and specialities.

**Methods:**

We analysed the 2025 GMC NTS post-speciality-by-site extract for medicine, benchmarked against all medical speciality posts. Each row represented an aggregated site × speciality × indicator cell. The primary metric was a gap score, defined as the site mean minus the national mean. Analyses followed a prespecified descriptive framework: deanery × indicator profiling, indicator-level comparisons with effect sizes and false discovery rate correction, intraclass correlation coefficients (ICCs), site-level correlation and volatility analyses, a suppression audit, outcome-category comparison, and principal component analysis (PCA)/cluster analysis.

**Results:**

The NTS data included 26,190 scored observations across 16 deaneries, 31 medical specialities, and 18 indicators. The mean deanery gap ranged from +3.23 in the North East to −3.13 in the East of England. The North East scored above the national average in 17 of 18 indicators, with the largest positive gaps in regional teaching (+7.34), rota design (+4.55), and reporting systems (+4.44). The East of England showed the largest negative gap in study leave (−10.17). After correcting for multiple testing, 17 of 18 indicators differed across deaneries; local teaching was the only indicator that did not differ. The largest deanery-level effect sizes were noted for regional teaching, clinical supervision out of hours, teamwork, and reporting systems. However, ICCs were modest overall, with the highest ICC observed for regional teaching (0.0973), indicating that a majority of the variance remained outside deanery-level clustering. Adequate experience correlated strongly with overall satisfaction (Spearman *ρ* = 0.848). Study leave showed the greatest site-to-site variation [standard deviation (SD) = 17.82]. Suppression for cells with fewer than three responses affected 21,060 of 47,442 rows (44.4%). Official NTS outcome categories also differed across deaneries (*χ*^2^ = 597.9, df = 60, *p* < 0.001).

**Conclusion:**

Training experience in UK medicine varies across deaneries and between individual sites. The findings support a dual improvement approach—deanery-wide attention to domains with stronger regional patterning, such as regional teaching and out-of-hours clinical supervision, alongside site-level review of highly variable domains such as study leave and rota design. PCA clustering and the resilience index should be interpreted as hypothesis-generating. Interpretation is also limited by the use of aggregated survey data and substantial suppression due to small cell counts.

## Introduction

Postgraduate medical education in the UK is delivered through rotational clinical posts across NHS trusts/boards and training sites. A deanery or equivalent postgraduate training body provides regional organisation, quality management, and oversight, whereas the individual site is the hospital or clinical location where day-to-day clinical work, supervision, teaching, and workplace learning are delivered. This structure indicates that poor or strong training experiences may arise from regional policy and governance, local site implementation, speciality-specific pressures, or a combination of these levels.

Postgraduate medical education is delivered predominantly through day-to-day clinical work, where the clinical learning environment shapes competence acquisition, professional identity, motivation, and retention (1). Supervision remains a core mechanism through which trainees gain safe autonomy, receive feedback, and translate clinical exposure into learning (2). Practical barriers, including time pressure, workload, competing service demands, and variable organisational support, can erode supervision quality and consistency (3).

The quality of trainees’ working and learning conditions has implications for patient safety and workforce sustainability. Systematic reviews report associations between clinician wellbeing, burnout, and safety-related outcomes, including perceived safety incidents and poorer quality-of-care indicators (4, 5). Burnout is common during postgraduate training across specialities and countries (6, 7). Physician burnout has also been associated with reduced career engagement, which supports the need for organisational responses rather than a narrow focus on individual resilience (8). Training quality and working conditions, including job autonomy and structured support, have been associated with junior doctors’ intentions to leave clinical practice (9). UK graduate follow-up surveys similarly describe variation in early-career doctors’ perceived support and training quality (10).

More recent UK evidence reinforces why National Training Survey indicators should be interpreted as system-level signals. Trainee perceptions of supervision, rota design, teamwork, and satisfaction have been correlated with excess mortality at the NHS organisational level (11), and a single-centre rota redesign report suggests that the rota structure can be modified to improve equity and staffing processes (12). Survey work among British doctors has also highlighted rota issues, training pressures, leave, and work–life balance as barriers to home-life satisfaction (13).

The GMC NTS is an established UK-wide survey used to monitor and benchmark postgraduate training experience (14, 15). The 2025 GMC report describes the survey as a large national dataset completed by approximately 71,000 doctors and highlights continuing concerns about workload, burnout, and escalation culture (16). The GMC education data tool provides trainee indicator outputs at multiple aggregation levels, including post-speciality by site, deanery/National Health Service England (NHSE) local office, trust/board, and site (17). Previous studies have examined speciality-specific variation and quality improvement applications using NTS-derived data; however, it is necessary to distinguish deanery-level patterns from site-driven variation when interpreting medical speciality results (18–21).

The research question for this study is as follows: Within UK medical speciality posts, which aspects of 2025 NTS variation are more consistent with deanery-level patterns, and which appear predominantly site- or speciality-driven? The specific objectives were to (1) describe deanery-level and speciality-level gap profiles; (2) quantify indicator-specific deanery differences using effect sizes; (3) estimate the proportion of variance attributable to deanery membership using ICCs; (4) identify indicators that co-vary across sites and those with high site-to-site volatility; (5) assess the extent and distribution of small-number suppression; and (6) present PCA/clustering and a resilience index.

## Methods

### Data source and study design

We used the 2025 GMC NTS post-speciality-by-site extract for medicine, benchmarked against all medical speciality posts (17). Rows in the extract corresponded to aggregated site × speciality × indicator combinations rather than individual trainee-level records. The Defence Postgraduate Medical Deanery and unspecified deanery were excluded before the analytic deanery comparisons. For each scored observation, a gap score was computed as follows: gap = site mean – national mean (all medical speciality posts benchmark). Scores are reported on a 0–100 scale. Since the available analytic unit was an aggregated survey cell, the study was designed as an ecological, descriptive analysis and not as an individual-level causal or multilevel modelling study.

### Units of analysis and included groups

A total of 16 deaneries were included in the analysis. A total of 18 NTS indicators were analysed: adequate experience, clinical supervision, clinical supervision out of hours, educational governance, educational supervision, facilities, feedback, handover, induction, local teaching, overall satisfaction, regional teaching, reporting systems, rota design, study leave, supportive environment, teamwork, and workload. Speciality-level profiling, PCA, clustering, within-deanery inequality, and speciality heterogeneity analyses were restricted to 16 major medical specialities with at least 50 scored observations.

### Analytical framework and rationale

First, deanery-level summaries and deanery × indicator heatmaps described where mean gaps were above or below the national benchmark. Second, indicator-level comparisons tested whether gap distributions differed across deaneries. The Kruskal–Wallis tests were used as the primary non-parametric comparison because normality of aggregated gap distributions could not be assumed; a one-way analysis of variance (ANOVA) was reported as a complementary parametric sensitivity analysis (22–24). *p*-values across the 18 indicators were adjusted using the Benjamini–Hochberg false discovery rate procedure (25).

Third, effect sizes were prioritised to avoid overinterpreting statistical significance in this large dataset. For *η*^2^ and *ω*^2^, values of approximately 0.01, 0.06, and 0.14 were interpreted as small, medium, and large effects, respectively; for Mann–Whitney *r*, values of approximately 0.10, 0.30, and 0.50 were interpreted as small, medium, and large effects (26). These thresholds were used as interpretative guides. Fourth, ICCs were computed for each indicator to estimate the proportion of site-level variance attributable to deanery membership, with an n_0_ adjustment for unequal deanery sizes (27). ICCs were interpreted as variance-partitioning descriptors. Fifth, for the 247 sites with complete data across all 18 indicators, site-level means were calculated for each indicator, and Spearman’s *ρ* and Pearson’s *r* correlations were estimated for all 153 indicator pairs. This analysis was intended to identify domains that tended to move together across sites. Site-to-site volatility was summarised using SD and interquartile range (IQR) of site-level gap scores for each indicator.

Sixth, suppression was examined because GMC outputs do not report scores for cells with fewer than three responses. Suppression rates were summarised overall and by deanery, speciality, indicator, and deanery × indicator. Outcome categories (below, Q1-not-below, within IQR, Q4-not-above, above) were compared across deaneries using Pearson *χ*^2^ tests, with Cramér’s *V* used as an effect size descriptor (28). Finally, the resilience index and PCA/clustering analyses were retained as exploratory summaries. The resilience index combined the breadth of above-national indicators with consistency across indicators and should not be interpreted as a validated psychometric scale. PCA/clustering was used for descriptive pattern recognition only; components are ordered by explained variance by definition, and cluster labels were not externally validated (29, 30).

## Results

### Deanery-level performance

The analytic dataset comprised 26,190 scored observations across 16 deaneries, 31 medical specialities, and 18 indicators (Table 1). Deanery sample size ranged from 613 scored observations [the Northern Ireland Medical and Dental Training Agency (NIMDTA)] to 3,353 (North West). The mean deanery score ranged from 67.27 in the East of England to 73.77 in the North East. Across all indicators and specialities, the mean deanery gap ranged from +3.23 (North East) to −3.13 (East of England), a spread of 6.36 points.

**Table 1:** Deanery-level summary statistics. Gap was calculated as the site mean minus the national mean for all medical speciality posts. The *N* score indicates the number of unsuppressed scored site × speciality × indicator observations included after exclusions.

Deanery	Sites	Specialities	*N* (scored)	Mean score	Mean gap	SD gap	Median gap
North East	18	17	1,477	73.77	+3.23	11.11	4.06
North West London	11	16	1,076	71.71	+1.43	12.03	2.62
NIMDTA	13	16	613	71.76	+1.37	10.88	1.74
NES	31	19	2039	71.70	+1.34	11.23	1.96
South London	17	19	1,536	71.44	+1.14	11.15	1.64
North Central and East London	16	22	1781	71.30	+0.99	11.65	1.54
North West	36	20	3,353	70.82	+0.56	10.91	1.14
South West	18	18	1915	70.78	+0.39	10.45	1.05
Thames Valley	8	18	702	70.53	+0.09	10.25	1.11
Wessex	12	17	1,185	70.47	+0.07	10.69	0.35
West Midlands	22	19	2,102	69.75	−0.82	10.06	−0.27
Health Education and Improvement Wales	20	14	1,075	68.56	−1.79	12.18	−1.70
Kent, Surrey, and Sussex	20	17	2053	68.46	−1.93	10.18	−1.55
East Midlands	15	19	1,480	67.74	−2.74	11.50	−2.20
Yorkshire and the Humber	22	19	1841	67.52	−2.90	11.59	−1.71
East of England	18	17	1962	67.27	−3.13	10.56	−2.50

### Deanery × indicator profile: breadth versus domain-specific deficits

**Figure 1:**
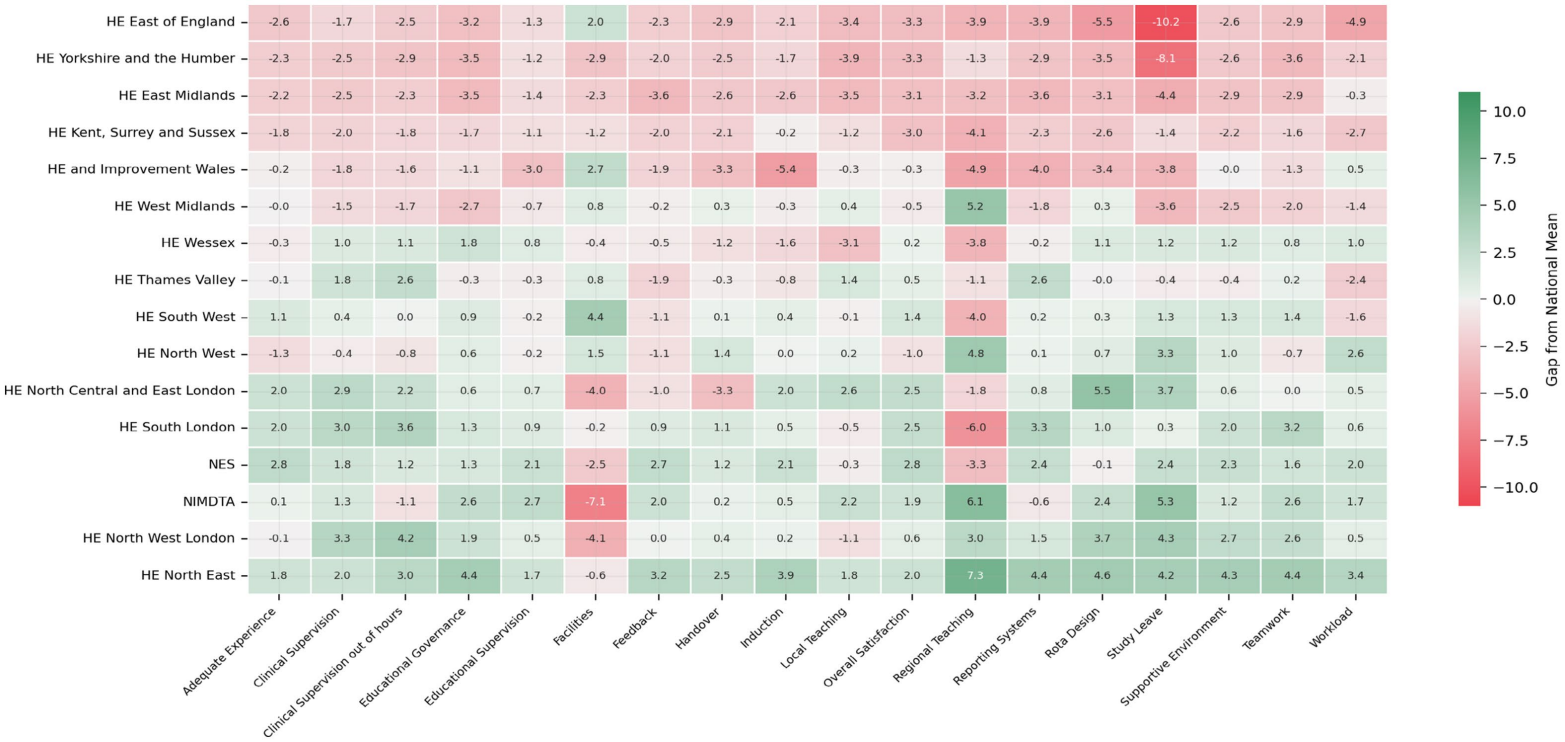
Deanery × indicator heatmap. Each cell shows the mean gap score for a deanery and indicator, calculated as the site mean minus the national benchmark. Green indicates an above-national-mean gap, and red indicates a below-national-mean gap.

The 16 × 18 deanery indicator matrix showed broad variation across domains (Figure 1). The North East was positive on 17 of 18 indicators, with particularly large gaps in regional teaching (+7.34), rota design (+4.55), and reporting systems (+4.44). The East of England showed the largest negative gap for study leave (−10.17). Yorkshire and the Humber; the East Midlands; and Kent, Surrey, and Sussex were below the national benchmark across all 18 indicators.

### Speciality-level signals

**Figure 2:**
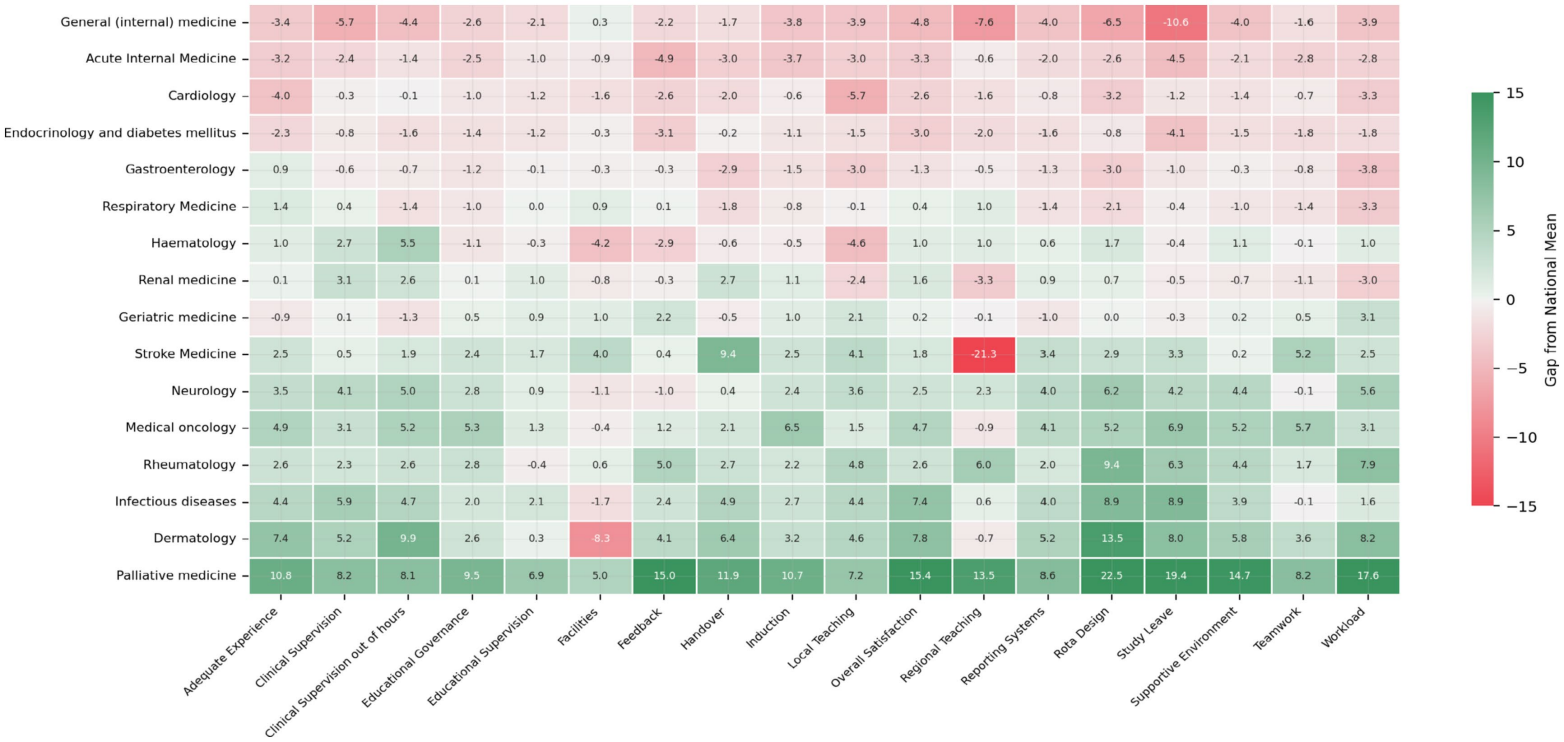
Speciality training domain fingerprints. Each cell shows the mean gap from the national benchmark for a speciality and an indicator among the 16 major medical specialities with at least 50 scored observations. Rows are sorted by the overall average gap.

Among the 16 major specialities (≥50 scored observations), speciality-indicator fingerprints showed wide dispersion and distinct profiles (Figure 2). Palliative medicine was above the national benchmark for all indicator means, with gaps ranging from +5.01 (facilities) to +22.49 (rota design). General (internal) medicine showed predominantly negative gaps (17 of 18 indicators), including rota design (−6.51), overall satisfaction (−4.80), and study leave (−10.56); its only positive indicator mean was facilities (+0.32). Stroke medicine demonstrated a mixed but often adverse pattern, including a large negative mean for regional teaching (−21.31).

### Deanery effects by indicator

**Figure 3:**
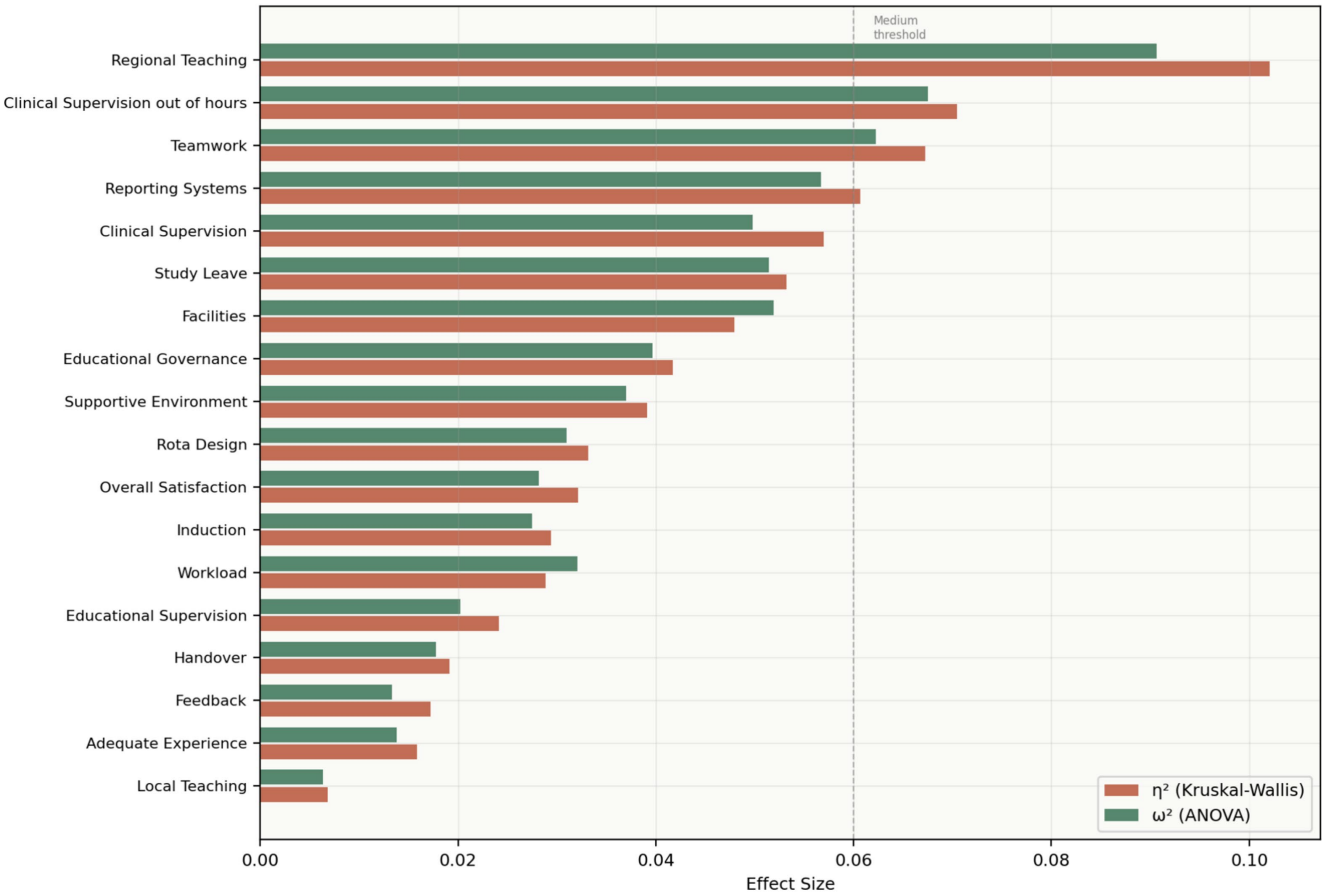
Indicator-level effect sizes for deanery differences. Bars show non-parametric *η*^2^ and parametric *ω*^2^. The dashed reference line marks the conventional medium-effect threshold of approximately 0.06; thresholds are interpretative guides rather than educational cutoffs.

After false discovery rate correction across 18 indicators, 17 indicators showed statistically significant differences in gap distributions across deaneries (Table 2); local teaching did not show statistically significant differences in gap distributions across deaneries. Interpretation was based on effect size and statistical significance. The largest deanery effects (Figure 3) were observed for regional teaching (*H* = 129.66, *η*^2^ = 0.1021; *ω*^2^ = 0.0906), clinical supervision out of hours (*η*^2^ = 0.0704; *ω*^2^ = 0.0675), teamwork (*η*^2^ = 0.0672; *ω*^2^ = 0.0622), and reporting systems (*η*^2^ = 0.0607; *ω*^2^ = 0.0567).

**Table 2:** Deanery effects by indicator. H denotes the Kruskal–Wallis statistic; *η*^2^ denotes eta-squared; *F* denotes the one-way ANOVA statistic; *ω*^2^ denotes omega-squared; BH adj denotes the Benjamini–Hochberg adjusted *p*-value. Effect sizes should be interpreted as magnitude descriptors rather than proof of causal deanery effects.

Indicator	*N*	*H*	*p* (BH adj)	*η* ^2^	*F*	*p* (ANOVA BH adj)	*ω* ^2^
Regional teaching	1,139	129.66	<0.0001	0.1021	8.57	<0.0001	0.0906
Clinical supervision out of hours	1,441	115.35	<0.0001	0.0704	7.96	<0.0001	0.0675
Teamwork	1,586	120.52	<0.0001	0.0672	8.02	<0.0001	0.0622
Reporting systems	1,457	102.45	<0.0001	0.0607	6.84	<0.0001	0.0567
Clinical supervision	1,557	102.78	<0.0001	0.0570	6.44	<0.0001	0.0498
Study leave	1,337	85.29	<0.0001	0.0532	5.83	<0.0001	0.0514
Facilities	1,283	75.76	<0.0001	0.0480	5.68	<0.0001	0.0519
Educational governance	1,587	80.55	<0.0001	0.0417	5.37	<0.0001	0.0397
Supportive environment	1,588	76.52	<0.0001	0.0391	5.06	<0.0001	0.0370
Rota design	1,556	66.12	<0.0001	0.0332	4.32	<0.0001	0.0310
Overall satisfaction	1,588	65.55	<0.0001	0.0322	4.07	<0.0001	0.0282
Induction	1,588	61.21	<0.0001	0.0294	3.99	<0.0001	0.0275
Workload	1,588	60.34	<0.0001	0.0288	4.51	<0.0001	0.0321
Educational supervision	1,588	52.90	<0.0001	0.0241	3.18	<0.0001	0.0202
Handover	1,253	38.64	0.0008	0.0191	2.51	0.0014	0.0178
Feedback	1,327	37.61	0.0011	0.0172	2.19	0.0056	0.0133
Adequate experience	1,588	39.96	0.0006	0.0159	2.48	0.0015	0.0138
Local teaching	1,139	22.62	0.0925	0.0068	1.48	0.1040	0.0063

### Between-deanery variance by intraclass correlation coefficient

**Figure 4:**
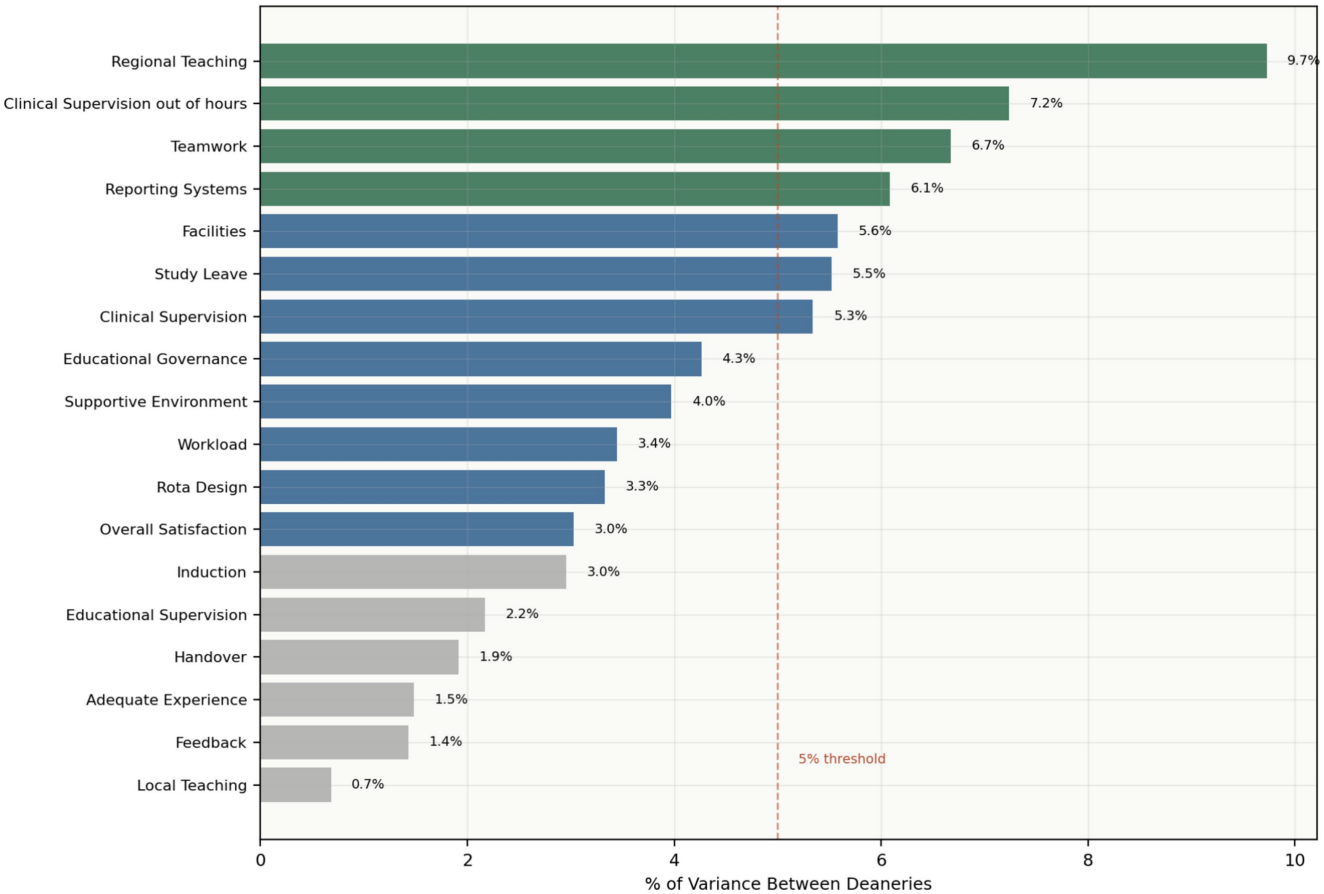
Intraclass correlation coefficient (ICC) by indicator. Higher values indicate a greater proportion of site-level variance attributable to deanery membership. The dashed 5% line is a pragmatic visual reference; ICCs should be interpreted as variance descriptors, not causal effects.

ICC values showed that only a modest proportion of total site-level variance was attributable to deanery membership for most indicators (Figure 4). The highest ICC was observed for regional teaching (0.0973; approximately 9.7% of variance between deaneries), followed by clinical supervision out of hours (0.0724), teamwork (0.0667), and reporting systems (0.0609). Lower ICC values were observed for domains described as more site-driven, including local teaching (0.0068), feedback (0.0143), and adequate experience (0.0148).

### *Post-hoc* extreme contrasts

Extreme-group contrasts were used to illustrate the magnitude of separation between the three highest and three lowest deaneries for four prespecified indicators; these contrasts were not intended to replace the full indicator-level analyses. All four indicators showed statistically significant bottom-versus-top differences with small-to-moderate effect sizes: study leave: the East of England (mean gap −10.17) versus NIMDTA (+5.31) (*U* = 718.0, *p* < 0.001, *r* = 0.532); rota design: the East of England (−5.46) versus the North Central and East London (+5.45) (*p* < 0.001, *r* = 0.430); clinical supervision: the East Midlands (−2.54) versus North West London (+3.35) (*p* < 0.001, *r* = 0.442); and overall satisfaction: the East of England (−3.28) versus NHS Education for Scotland (NES) (+2.79) (*p* < 0.001, *r* = 0.336).

### Indicator coupling and volatility

**Figure 5:**
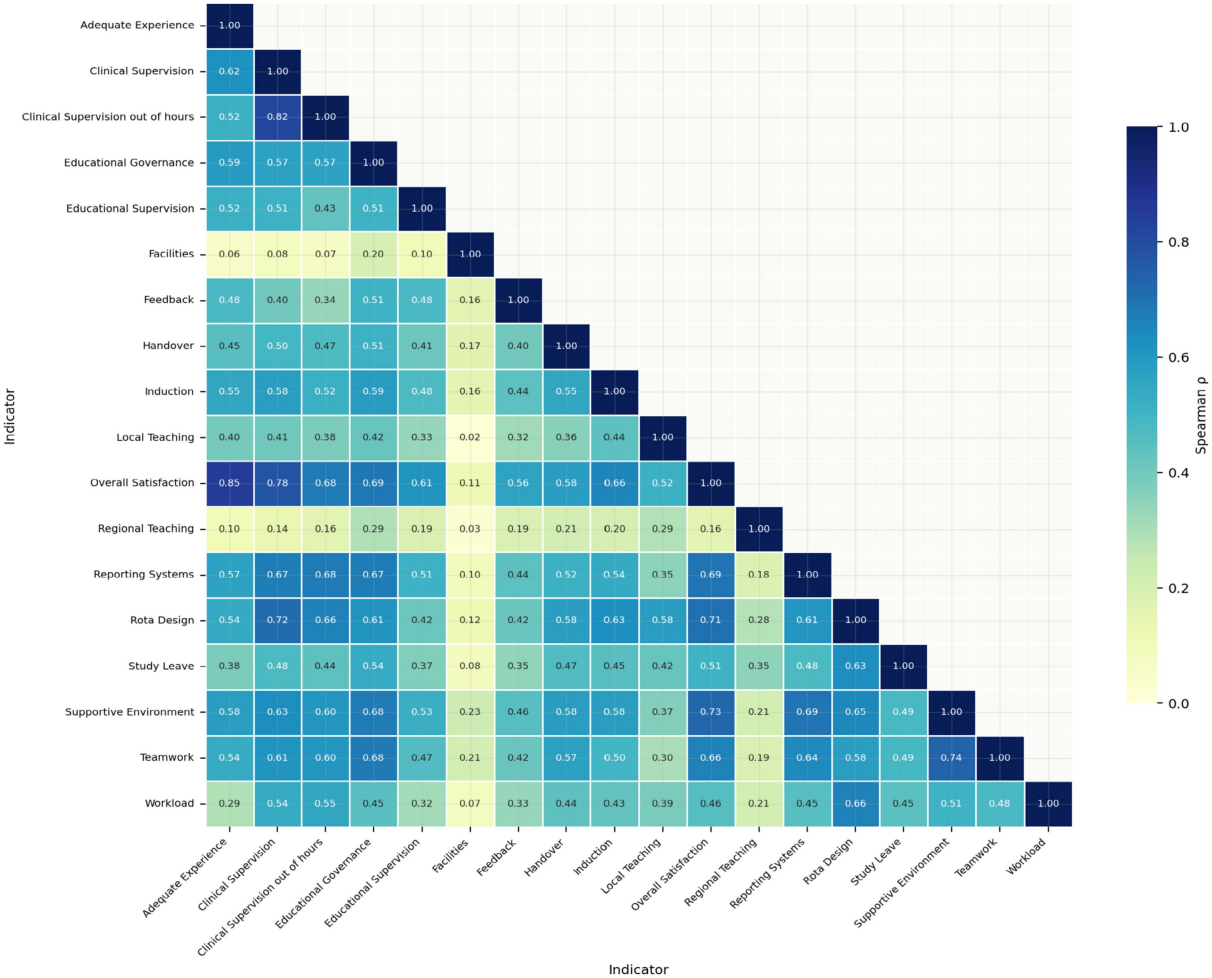
Indicator coupling across the sites. The lower-triangle matrix shows Spearman’s correlation coefficients between site-level indicator means for the 247 sites with complete data across all 18 indicators. Darker shading indicates a stronger positive correlation; correlations do not establish directionality.

**Figure 6:**
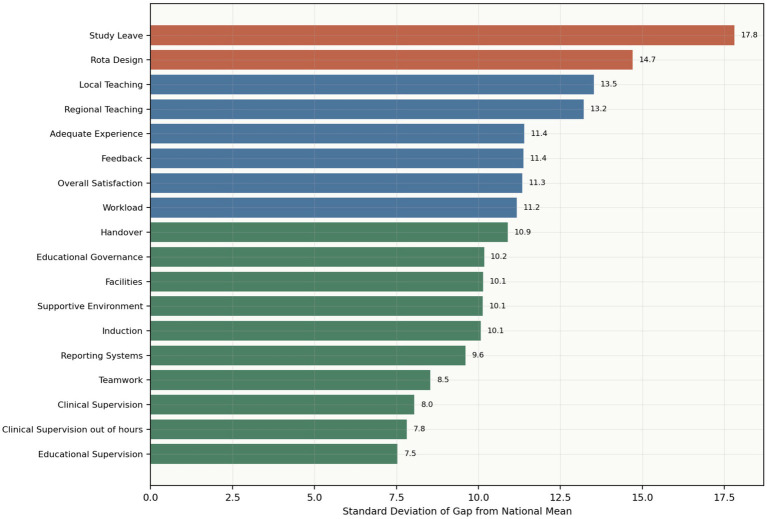
Indicator volatility ranking. Volatility was quantified using the standard deviation of site-level gap scores for each indicator. Red indicates the most site-variable indicators, blue indicates intermediate variability, and green indicates the most stable indicators.

A correlation analysis used the 247 sites with complete data for all 18 indicators (Figure 5). The strongest positive Spearman correlations were adequate experience with overall satisfaction (*ρ* = 0.848; Pearson *r* = 0.845), clinical supervision with clinical supervision out of hours (*ρ* = 0.818), clinical supervision with overall satisfaction (*ρ* = 0.781), and supportive environment with teamwork (*ρ* = 0.737). The weakest couplings involved facilities and teaching metrics, including facilities with local teaching (*ρ* = 0.023) and facilities with regional teaching (*ρ* = 0.027). Indicator volatility rankings (Figure 6) identified study leave as the most site-variable domain (SD = 17.82), followed by rota design (SD = 14.71) and local teaching (SD = 13.53). Supervision domains were comparatively stable (educational supervision, SD = 7.54; clinical supervision, SD = 8.05).

### *N* < 3 suppression

**Figure 7:**
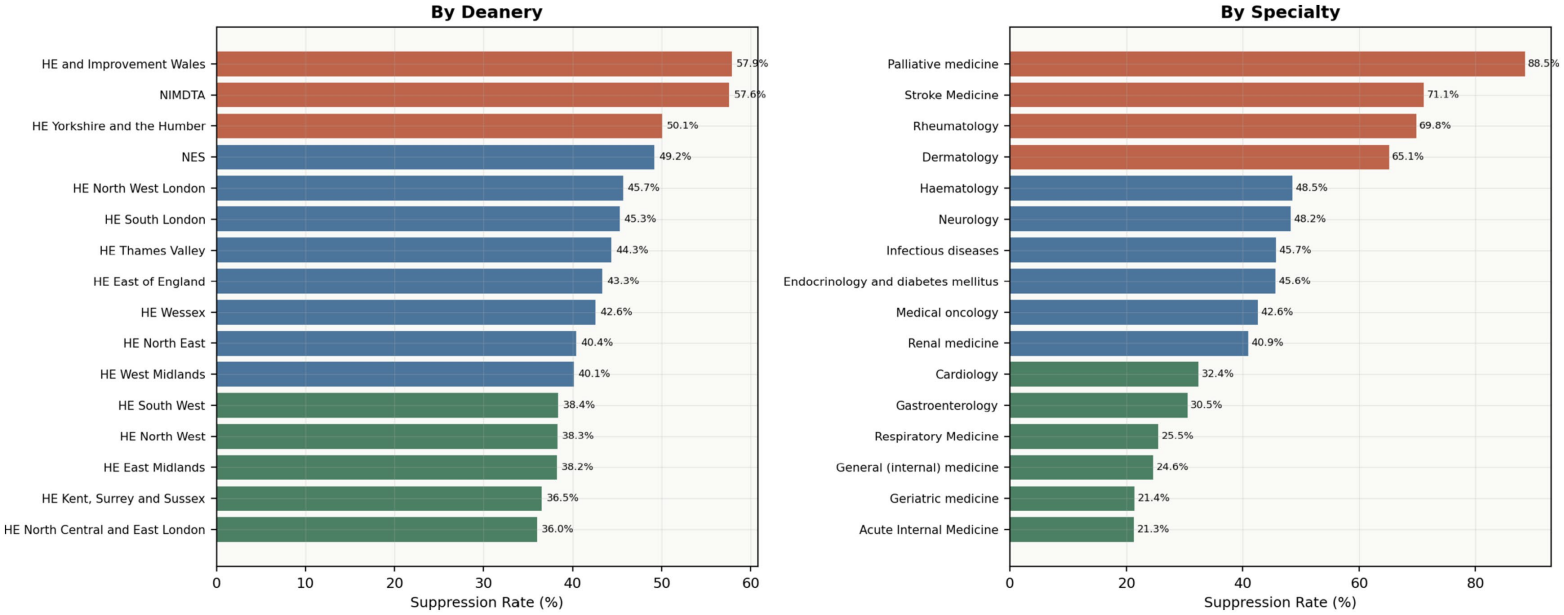
Small-number suppression by deanery and speciality. Suppression indicates cells with fewer than three responses for which a score was not reported. Higher suppression may reduce the visibility of performance in smaller deaneries, specialities, or site-speciality cells.

Suppression affected 21,060 of 47,442 medicine rows (44.4%) in the extract (Figure 7). After the prespecified exclusion of the Defence Postgraduate Medical Deanery and an unspecified deanery, the data used for deanery comparisons comprised 26,190 scored observations. Suppression was highest in Health Education and Improvement Wales (HEIW) (57.9%) and NIMDTA (57.6%). Speciality-level suppression was very high for palliative medicine (88.5%), stroke medicine (71.1%), and rheumatology (69.8%). Teaching indicators showed the highest suppression rates, including regional teaching and local teaching (each 52.2%), whereas clinical supervision out of hours had the lowest suppression rate (39.1%).

### Outcome distributions

**Figure 8:**
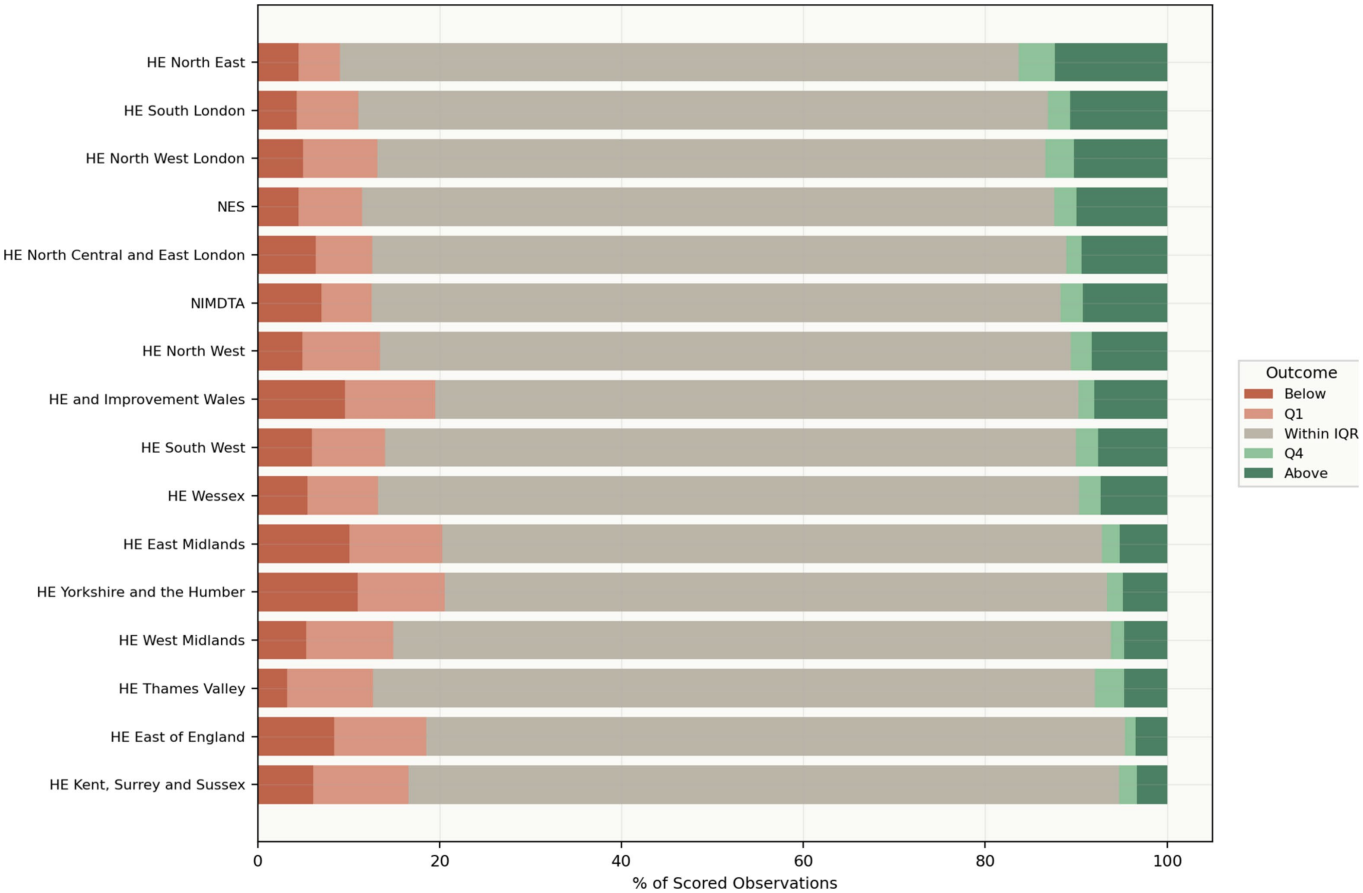
Stacked NTS outcome distribution by deanery. Bars show the proportion of scored observations in each official NTS outcome category: below, Q1-not-below, within IQR, Q4-not-above, and above. The comparison is descriptive and is based only on unsuppressed scored observations.

Outcome classification distributions differed by deanery (Figure 8) (*χ*^2^ = 597.9, df = 60, *p* < 0.001), with Cramér’s *V* = 0.0755. Notable contrasts included the North East, where 12.32% of scored observations were classified as above and 4.47% as below (above-below ≈ + 7.85 percentage points); Yorkshire and the Humber, where 10.97% were below and 4.89% above (above-below ≈ − 6.08 percentage points); and the East of England, which had the lowest above rate (3.47%).

**Figure 9:**
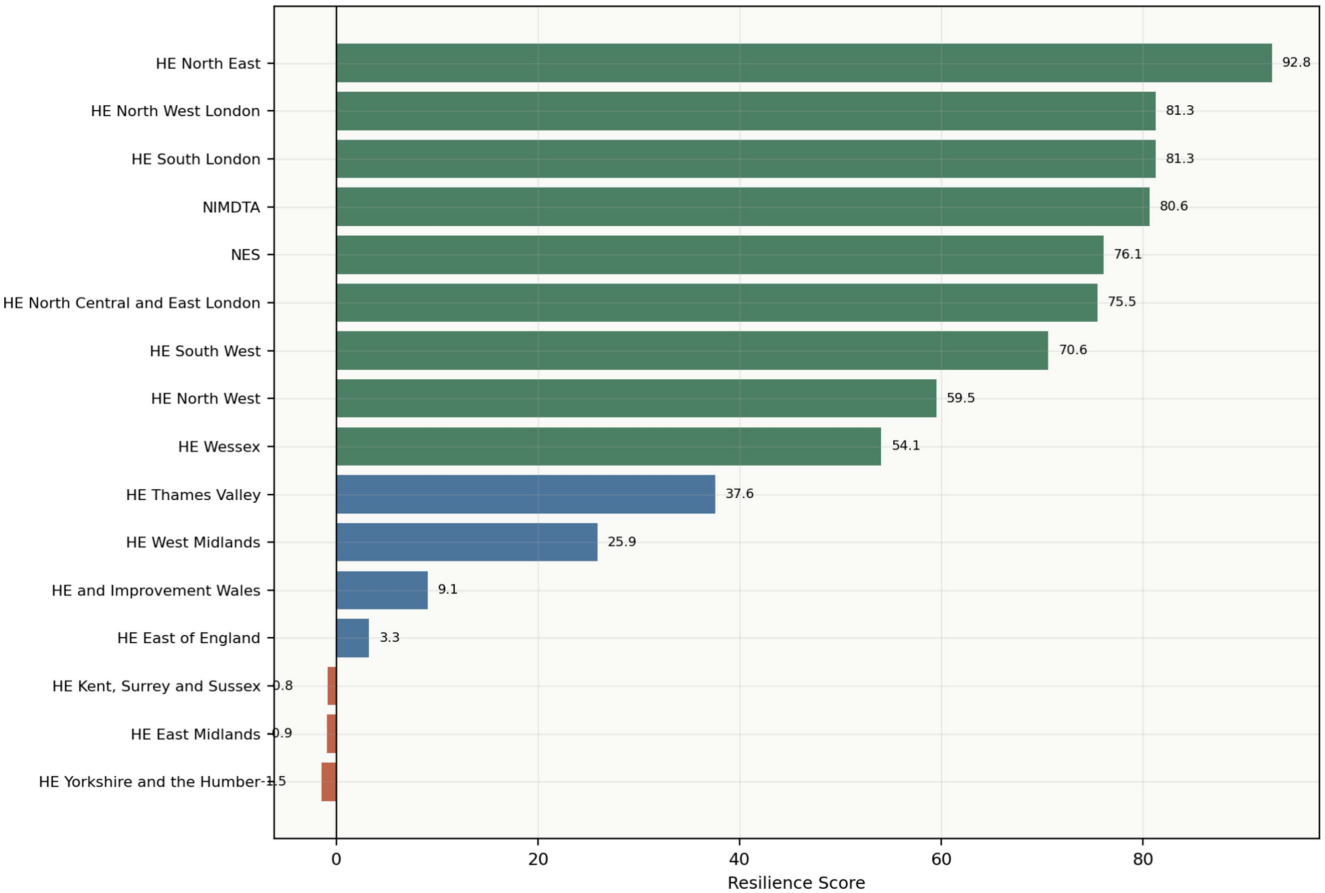
Exploratory resilience score by deanery. The score combines the percentage of indicators above the national benchmark with consistency across indicators. Green, blue, and red indicate higher, intermediate, and lower values, respectively. This composite is exploratory and not a validated performance scale.

The resilience index was treated as a composite summary rather than a validated outcome measure (Figure 9). The North East had the highest resilience score (92.7), corresponding to 17 of the 18 indicators above the national benchmark. Three deaneries had negative resilience scores: Kent, Surrey, and Sussex (−0.8); the East Midlands (−0.9); and Yorkshire and the Humber (−1.5).

PCA and clustering analyses were exploratory and hypothesis-generating (Supplementary Figures S1, S2). Speciality PCA found that PC1 accounted for 77.08% of the variance explained, with smaller contributions from PC2 (9.16%) and PC3 (6.21%). Since PCA components are ordered by explained variance, this distribution was interpreted descriptively rather than as a separate finding. Silhouette scores supported *k* = 3–4 cluster solutions (*k* = 3: 0.401; *k* = 4: 0.390; *k* = 5: 0.234). Deanery PCA found that PC1 accounted for 66.53% of the variance explained and PC2 accounted for 9.03% of the variance explained.

### Within-deanery inequality and speciality heterogeneity

Within-deanery cross-speciality inequality was relatively uniform across deaneries (average SD range: 9.77–11.79), with Health Education and Improvement Wales being the highest (11.79) and the Thames Valley being the lowest (9.77). Inequality was most pronounced in study leave (average SD 17.09) and rota design (average SD 14.17). Across specialities, heterogeneity (average cross-site SD) was highest for rheumatology (12.16) and lowest for palliative medicine (8.53). General (internal) medicine showed lower heterogeneity (9.40) despite consistently low mean gaps.

### Two-way interaction (overall satisfaction)

**Figure 10:**
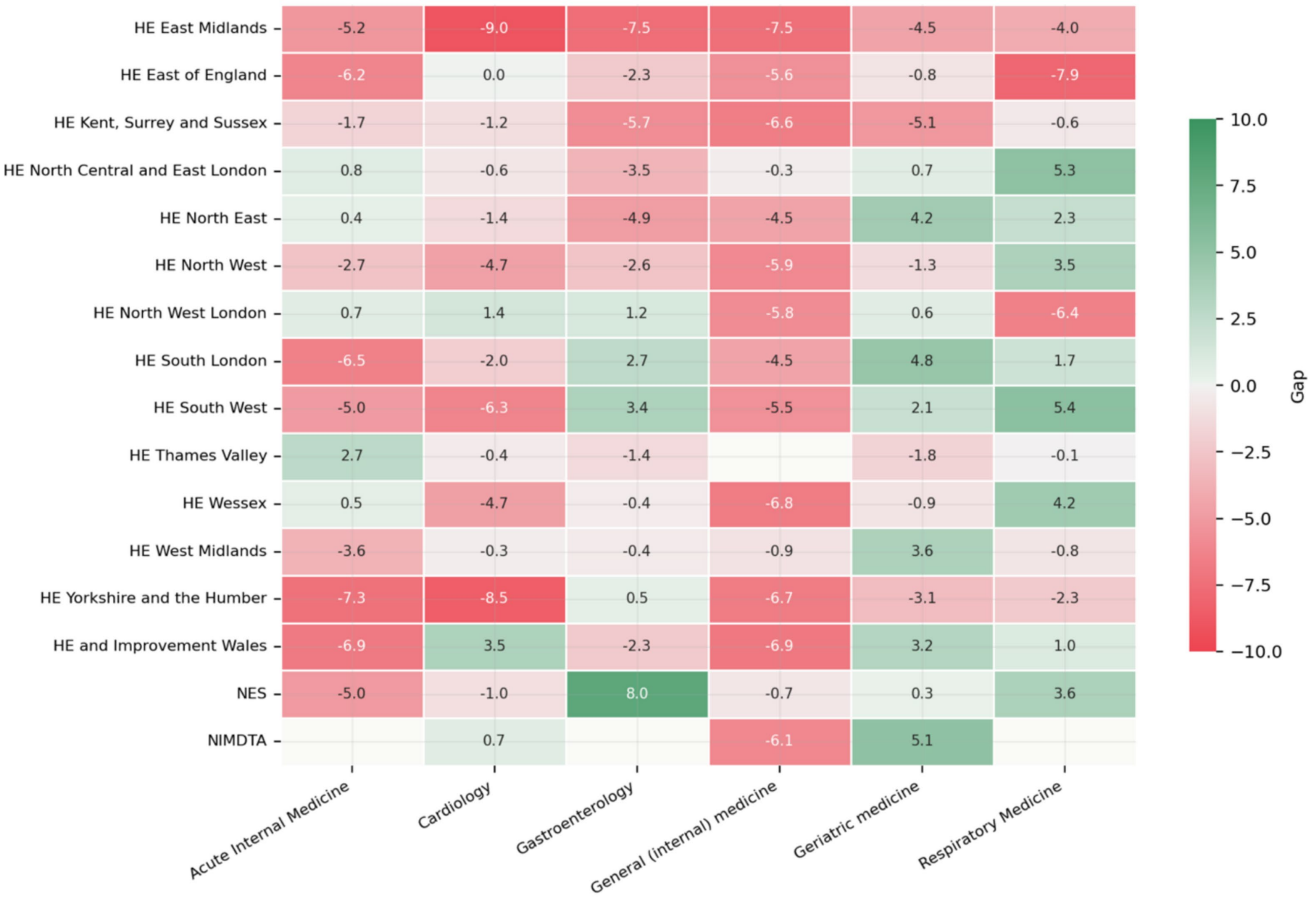
Two-way deanery × speciality heatmap for overall satisfaction. Cells show the mean overall satisfaction gap for six large specialities within deaneries, restricted to cells with *N* ≥ 3. Green indicates above the national gap, and red indicates below the national gap.

In the deanery × speciality analysis of overall satisfaction for six large specialities (cells restricted to *N* ≥ 3), general (internal) medicine was below the national benchmark in every deanery (range −0.3 to −7.5) and showed the least between-deanery SD (2.39). Respiratory medicine showed the greatest between-deanery variation (SD 4.09), indicating larger deanery dependence for this speciality’s overall satisfaction gaps (Figure 10).

## Discussion

This study used the 2025 GMC NTS post-speciality-by-site extract to examine whether variation in medicine training experience was more consistent with deanery-level patterns or local site/speciality variation. The main finding was not simply that deaneries differed, but that the degree of deanery-level patterning varied substantially by indicator. Regional teaching, clinical supervision out of hours, teamwork, and reporting systems showed the largest deanery effects, whereas local teaching, feedback, and adequate experience had low ICCs and appeared to be more site-driven.

The modest ICC values require careful interpretation. Even the highest ICC, for regional teaching, was 0.0973, indicating that a majority of variation remained outside deanery-level clustering. Therefore, statistically significant deanery differences should not be interpreted as evidence that deanery policy alone determines trainee experience. Instead, the findings support a dual-action model: Deanery-wide interventions may be appropriate where regional patterning is stronger, but site-level diagnostics remain necessary for domains with low ICCs or high local volatility.

The correlation and volatility analyses provide practical prioritisation signals but should not be interpreted causally. Adequate experience was strongly correlated with overall satisfaction, suggesting that trainees’ perceptions of sufficient clinical exposure may be a useful lead indicator of broader training sentiment. However, this association cannot determine whether adequate experience drives satisfaction, reflects the same underlying workplace conditions, or both. Study leave and rota design were prominent because they showed large site-to-site variability and clear extreme-group contrasts, suggesting that both regional policy and local implementation may influence these domains.

The results are consistent with wider UK literature indicating that supervision, rota design, teamwork, workload, and work–life balance are meaningful components of the training environment (11–13). The GMC NTS report also highlights continued concerns around workload, burnout, and trainee escalation culture (16). These data support the relevance of NTS indicators as system-level signals.

Study leave warrants particular attention because it had the greatest site-to-site variability in this analysis. Contemporary trainee survey work on learning experiences abroad describes perceived educational value, accessibility concerns, and funding barriers, which aligns with the interpretation that leave processes may affect perceived fairness and opportunity (31). Rota design is similarly actionable: Recent scheduling studies report associations between rota structures and resident wellness or fatigue, supporting rota design as a plausible organisational lever while recognising that these associations are context-dependent (32, 33). The suppression audit is important for interpreting the visibility of training quality. Approximately 44% of medicine rows were suppressed because fewer than three trainees responded. This may disproportionately reduce visibility in smaller specialities, smaller deaneries, and low-count site-speciality cells, potentially masking both excellence and concern. The present study summarised suppression by deanery, speciality, indicator, and deanery × indicator but did not perform additional suppression-performance correlation analyses. Such analyses should be performed in future studies if row-level denominators and sufficient unsuppressed data are available.

The PCA/clustering and resilience analyses were conducted as exploratory summaries. PCA is useful for visualising multivariate profiles; however, the ordering of components by explained variance is inherent to the method and should not be overinterpreted. Cluster groupings were not externally validated, and terms such as “tiers” have therefore been avoided in favour of cautious descriptive wording. Similarly, the resilience index is an *ad hoc* composite that may help summarise breadth and consistency, but it should not be treated as a validated measure of training quality.

### Strengths and limitations

The strengths of this study include the use of a national GMC dataset, a transparent gap score definition, correction for multiple testing, and consistent reporting of effect sizes alongside *p*-values. The analysis also adds ICC, coupling, volatility, and suppression summaries to make the results more interpretable for quality improvement rather than simple ranking.

The limitations are substantial. First, the study used aggregated site × speciality × indicator cells rather than individual trainee responses. Rows therefore cannot be assumed to represent independent trainees, and non-hierarchical tests may overstate statistical precision. Multilevel or mixed-effects modelling was not performed in this revision; future analyses with appropriate individual-level or weighted aggregated data should model trainees nested within sites, specialities, and deaneries. Second, the design is cross-sectional and perception-based, so associations cannot establish direction or causality. Third, a negative gap represents deviation from a national benchmark and should not be interpreted as an absolute unacceptable threshold. Fourth, small-number suppression caused substantial information loss and may cause bias visibility towards larger sites or specialities. Finally, exploratory PCA/clustering and the resilience index require external validation before being used for performance classification.

### Future directions

Future studies should evaluate whether targeted interventions in high-leverage domains, such as rota design, study leave processes, and out-of-hours supervision, improve subsequent NTS outcomes. Organisational interventions can reduce burnout, although effects vary by context (34). Qualitative follow-ups at outlier sites could clarify the operational mechanisms behind extreme gaps, and triangulation with objective measures such as rota gaps, teaching provision, exception reporting, and study-leave approval data would strengthen causal interpretation.

In summary, the study suggests that training experience in UK medicine varies across deaneries; however, much of the actionable variation remains at the site level. Separating where differences exist from where variance is concentrated provides a more cautious and practically useful interpretation of NTS data than ranking deaneries alone.

## Conclusion

This analysis of the 2025 GMC National Training Survey for medicine shows that trainees’ experiences vary across the UK, with some indicators showing modest deanery-level clustering and others appearing predominantly site-driven. Regional teaching, out-of-hours supervision, teamwork, and reporting systems showed the clearest deanery-level patterns, while study leave and rota design varied most between sites. The findings support coordinated deanery action for domains that reflect regional systems, alongside targeted site-level review where local implementation dominates. Interpretation should remain cautious because the analysis used aggregated cross-sectional survey data, did not include multilevel modelling, and was affected by substantial small-number suppression.

## Data Availability

The original contributions presented in the study are included in the article/Supplementary material, further inquiries can be directed to the corresponding authors.
