# Supplementary material for: Training experience across UK medicine: deanery differences versus site variation—General Medical Council National Training Survey 2025 analysis
Source: Front Med (Lausanne). 2026 Jun 24;13:1833862. doi: 10.3389/fmed.2026.1833862 (PMC13341737; doi:10.3389/fmed.2026.1833862)
Supplement: Supplementary file 1 [file Data_sheet_1.docx]

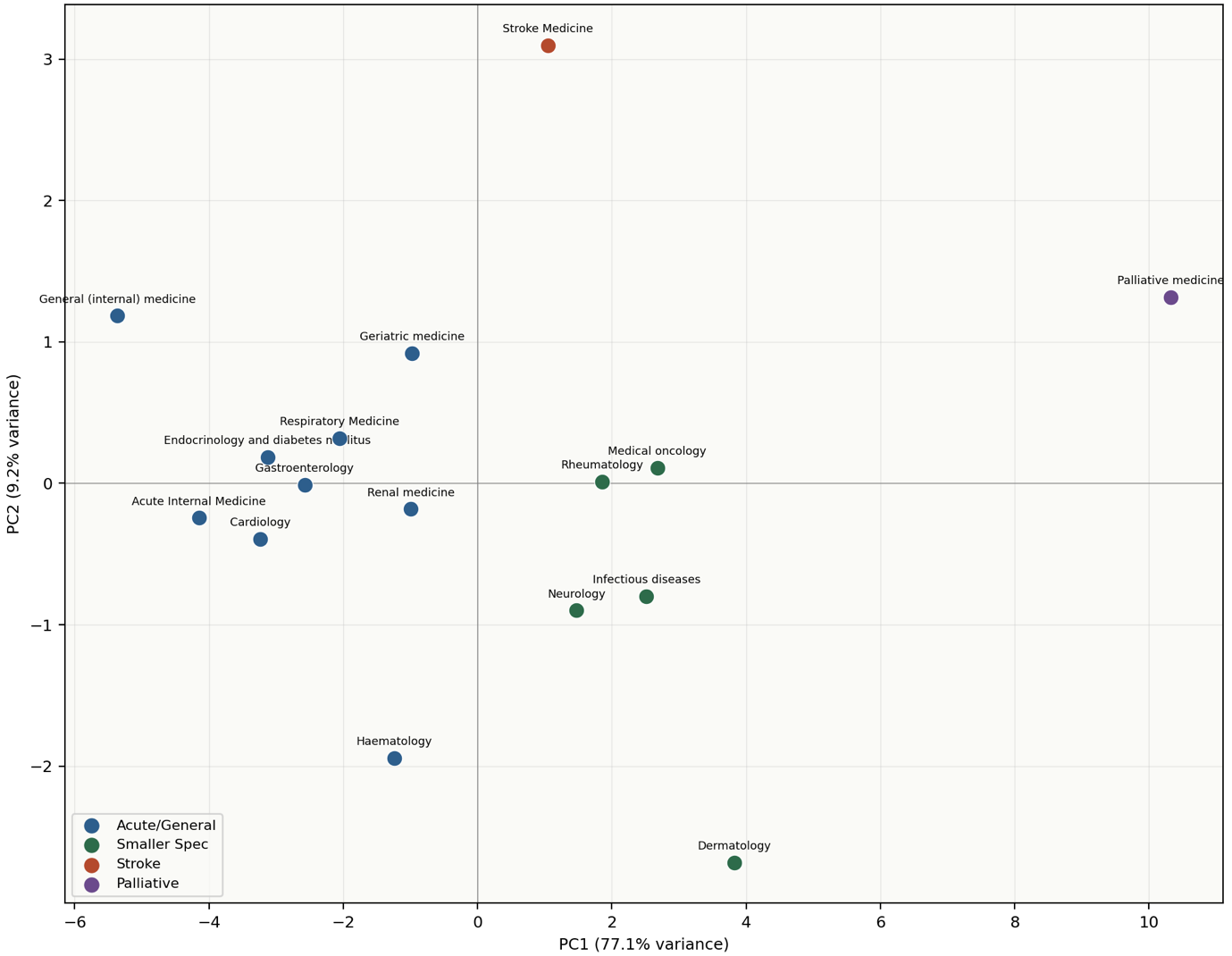


Supplementary Figure S1. PCA biplot of specialty profiles. The 16 × 18 specialty × indicator matrix was z-score standardised by indicator before PCA. The plot is intended to visualise multivariate similarity between specialties; cluster interpretation should be considered hypothesis-generating.


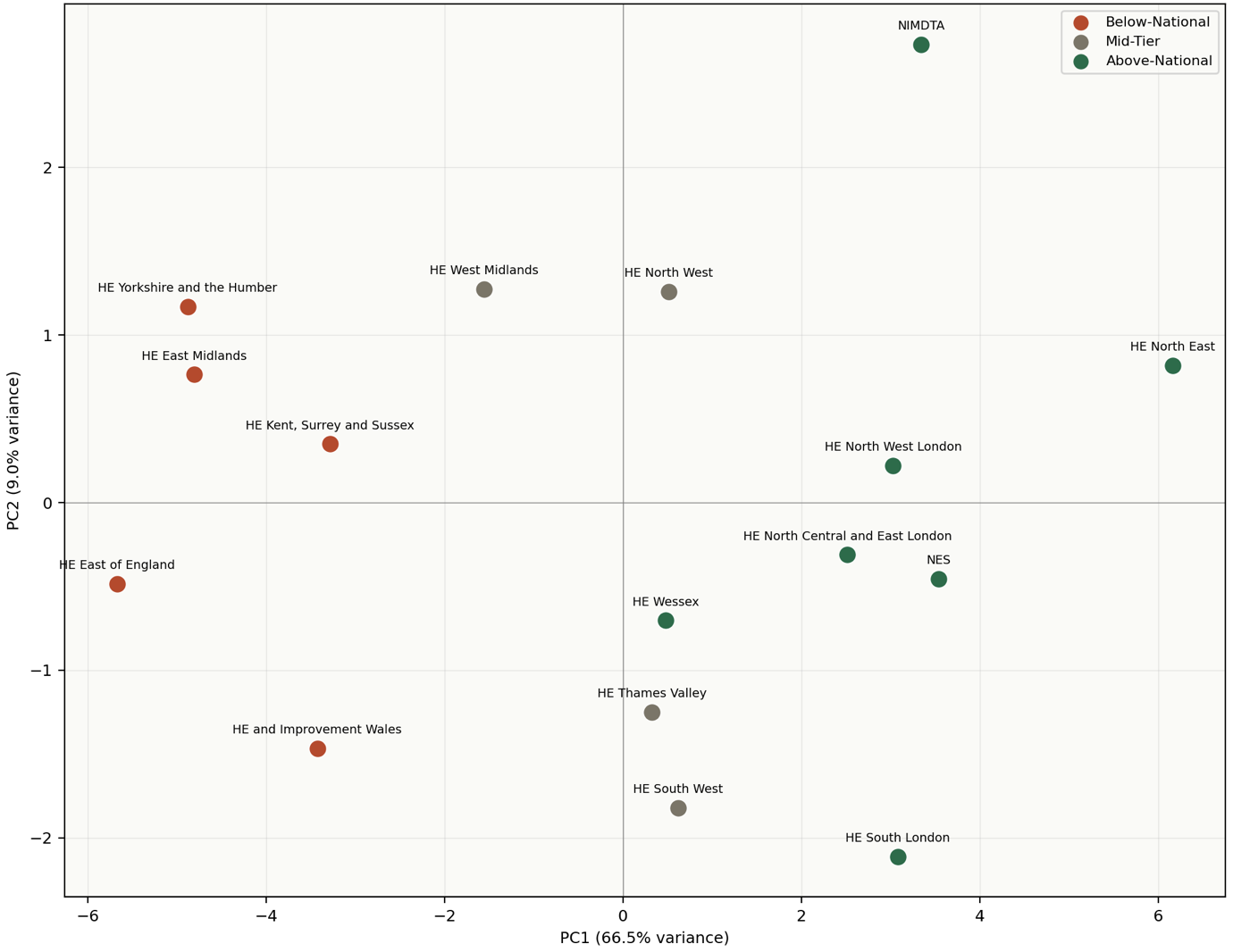


Supplementary Figure S2. PCA biplot of deanery profiles. The 16 × 18 deanery × indicator matrix was z-score standardised before PCA. Components are ordered by explained variance by definition, and cluster labels are not externally validated.
